# Multidimensional Sleep Health and Physical Functioning in Older Adults

**DOI:** 10.1093/geroni/igaa057.1382

**Published:** 2020-12-16

**Authors:** Caitlan Tighe, Ryan Brindle, Sarah Stahl, Meredith Wallace, Adam Bramoweth, Daniel Forman, Daniel Buysse

**Affiliations:** 1 VA Pittsburgh Healthcare System, Pittsburgh, Pennsylvania, United States; 2 Washington and Lee University, Lexington, Virginia, United States; 3 University of Pittsburgh, Pittsburgh, United States

## Abstract

Prior studies link specific sleep parameters to physical functioning in older adults. Recent work suggests the utility of examining sleep health from a multidimensional perspective, enabling consideration of an individual’s experience across multiple different sleep parameters (e.g., quality, duration, timing). We examined the associations of multidimensional sleep health with objective, performance-based measures of physical functioning in older adults. We conducted a secondary analysis of 158 adults (Mage=71.8 years; 51.9% female) who participated in the Midlife in the United States (MIDUS) 2 and MIDUS Refresher studies. We used data from daily diaries, wrist actigraphy, and self-report measures to derive a composite multidimensional sleep health score ranging from 0-6, with higher scores indicating better sleep health. Physical function was assessed using gait speed during a 50-foot timed walk, lower extremity strength as measured by a chair stand test, and grip strength assessed with dynamometers. We used hierarchical regression to examine the associations between sleep health and gait speed, lower extremity strength, and grip strength. Age, sex, race, education, depression symptoms, medical comorbidity, and body mass index were covariates in each model. In adjusted analyses, better multidimensional sleep health was significantly associated with faster gait speed (B=.03, p=.01). Multidimensional sleep health was not significantly associated with lower limb strength (B=-.12, p=.89) or grip strength (B=.45, p=.40). Gait speed is a key indicator of functional capacity as well as morbidity and mortality in older adults. Multidimensional sleep health may be a therapeutic target for improving physical functioning and health in older adults.

